# Evaluation of blood pressure variation in recovered COVID-19 patients at one-year follow-up: a retrospective cohort study

**DOI:** 10.1186/s12872-024-03916-w

**Published:** 2024-05-07

**Authors:** Pouria Azami, Reza Golchin Vafa, Reza Heydarzadeh, Mehrdad Sadeghi, Farhang Amiri, Alireza Azadian, Amin Khademolhosseini, Mina Yousefi, Mohammad Montaseri, Nazanin Hosseini, Seyed Ali Hosseini, Javad Kojuri

**Affiliations:** 1 Shiraz University of Medical Sciences, Shiraz, Iran; 2 Professor Kojuri Cardiology Clinic, Niayesh St. Niayesh Medical Complex, Shiraz, Iran; 3 Cardiology Department, Shiraz University of Medical Sciences, Shiraz, Iran; 4 Clinical Education Research Center, Shiraz University of Medical Sciences, Shiraz, Iran; 5 Shahid sadoughi University of Medical sciences, Yazd, Iran

**Keywords:** COVID-19, Hypertension, Blood pressure

## Abstract

**Background:**

Coronavirus disease 2019 (COVID-19) has various sequelae, one of which might be hypertension. We aimed to evaluate COVID-19’s impact on blood pressure (BP) in non-hospitalized patients at one-year follow-up.

**Method:**

A total of 7,950 consecutive COVID-19 patients regularly visiting our cardiology clinic were retrospectively screened. Patients’ electronic medical records including demographics, comorbidities, vital signs, treatments, and outcomes, were reviewed by two physicians. Individuals with at least one BP measurement in the three months preceding COVID-19 and one measurement in 12 months or more following recovery were included. BP levels before and after COVID-19 were compared using the paired t-test.

**Results:**

5,355 confirmed COVID-19 patients (mean age 55.51 ± 15.38 years) were included. Hypertension (56.9%) and diabetes mellitus (34%) were the predominant comorbidities, and 44.3% had prior major adverse cardiovascular events. Both systolic (126.90 ± 20.91 vs. 139.99 ± 23.94 mmHg, *P* < 0.001) and diastolic BP (80.54 ± 13.94 vs. 86.49 ± 14.40 mmHg, *P* < 0.001) were significantly higher post-COVID-19 vs. pre-COVID-19. Notably, 456 (14%) hypertensive patients experienced exacerbated hypertension, while 408 (17%) patients developed new-onset hypertension, overall 864 (16%) of patients had exacerbation or new hypertension. Linear regression analysis revealed that advanced age, smoking, previous cardiovascular events, hypertension, and diabetes mellitus predict increased BP following COVID-19 (*P* < 0.001).

**Conclusion:**

COVID-19 raised systolic and diastolic BP in the long term in non-hospitalized patients, with over one-sixth developing new-onset or exacerbated hypertension. All patients should be evaluated regarding BP, following COVID-19 recovery, particularly those with the mentioned predictive factors. (clinicaltrial.gov: NCT05798208)

## Introduction

The global impact of Coronavirus disease 2019 (COVID-19) has been staggering, with approximately seven million deaths reported as of February 2023 [1, 2]. As our understanding of the pathophysiology of this disease has evolved, various comorbidities have been investigated for their association with adverse COVID-19 outcomes. Among these, hypertension stands out as one of the most prevalent comorbidities, present in roughly 25% of COVID-19 patients [3]. Prior studies have explored its relationship with adverse COVID-19 outcomes including hospitalization, critical care, mechanical ventilation, and mortality [4–6].

With the pandemic’s incidence and mortality rates now relatively controlled, attention has shifted towards understanding the long-term consequences of COVID-19. Post-COVID-19 sequelae, such as persistent hypertension, poor diabetic control, major adverse cardiovascular events (MACE), and persistent renal damage, have emerged as areas of concern [7]. However, conflicting data exist regarding the pandemic’s impact on blood pressure (BP) control, with reports of both short-term increases [8, 9] and decreases [10, 11] in BP levels. Yet, there is limited knowledge about the medium and long-term effects of COVID-19 on BP [12]. In addition to these concerns, the emergence of Long COVID-19 Syndrome, characterized by a collection of persistent or new clinical manifestations appearing months after a COVID-19 infection, poses a new challenge in biomedical research [13]. The hallmark of this syndrome is prolonged endothelial dysfunction and microcirculation impairment, exacerbated by elevated levels of pro-inflammatory cytokines, including CXCL10 and IL-6 [14]. There is growing evidence to suggest that Long COVID-19 Syndrome could be recognized as a new risk factor for cardiovascular diseases [15, 16].

Given these considerations, this study aims to elucidate the long-term effects of COVID-19 on BP in non-hospitalized COVID-19 patients. We also seek to assess the predictors of BP variation and the prevalence of new-onset hypertension among COVID-19 patients.”

## Patients & methods

### Study design and participants

This single-center retrospective cohort study was conducted at an outpatient cardiology clinic in Shiraz, Iran, between February 15, 2020, and December 28, 2022. We evaluated patients with a confirmed diagnosis of severe acute respiratory syndrome coronavirus 2 (SARS-CoV-2) infection by RNA reverse-transcriptase polymerase-chain-reaction assays from nasopharyngeal or oropharyngeal swab specimens according to World Health Organization guidelines [17]. Two physicians (PA, MY) reviewed the patients’ electronic medical records, including demographics, comorbidities, vital signs, treatments, and outcomes. Ambiguities were resolved by a third author (MM).

**Fig. 1:**
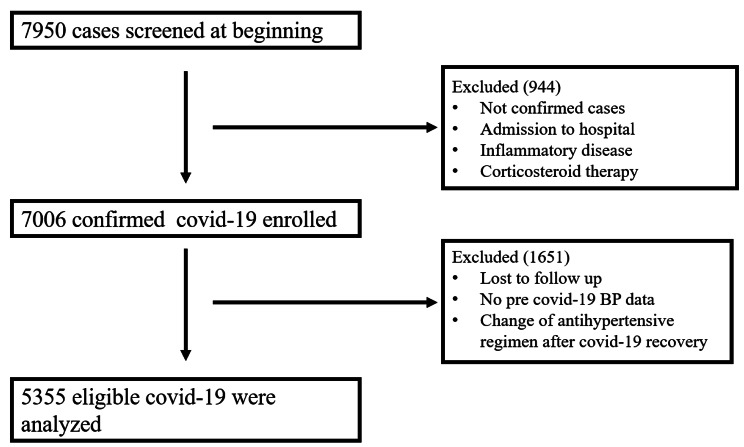
Flowchart of patient enrollment

The current study included all COVID-19 patients who had at least one documented blood pressure measurement in the three months preceding infection and one documented measurement twelve months or more following recovery. The following patients were excluded: those under 18, with severe COVID-19, which was defined as those who required hospitalization, admission to the intensive care unit (ICU), intubation, or mechanical ventilation due to COVID-19 [18], who received corticosteroid therapy, those with a history of systemic inflammatory disease, kidney or liver disease, immunocompromised patients, patients with persistent symptoms or long covid-19, and those who switched their antihypertensive regimen during the study. Finally, 5,355 eligible patients were investigated (Fig. 1). The primary outcome was to assess BP variation following infection (particularly a prolonged rise in BP) among non-hospitalized COVID-19 patients. The secondary outcome was to identify the predictor factors of BP variation.

The present study was approved by the Ethics Committee of Shiraz University of Medical Sciences under code IR.SUMS.MED.REC.1401.465, All patients were informed about the details of this research and provided their informed consent. For informed consent, we contacted the patients by telephone and described the details of the study and the anonymity of their data. Patients who declined to participate were excluded. All methods were performed in accordance with the Helsinki guidelines and regulations.

### Definitions

A follow-up systolic BP ≥ 140 or diastolic BP ≥ 90 mmHg requiring new or intensified antihypertensive therapy was defined as a prolonged rise in BP. Previous cardiac events included a history of heart failure (defined by at least one prior hospitalization for acute heart failure requiring intravenous therapy) and coronary artery disease (defined by at least one of the following criteria: 1- the presence of any epicardial coronary vessels with > 75% stenosis tested on coronary angiography; 2- history of an acute coronary syndrome; 3- coronary revascularization) [19]. Patients with an FEV1/FVC ratio below 0.7 at spirometry were considered to have chronic obstructive pulmonary disease (COPD). Cerebrovascular disease included patients with a history of transient ischemic attack (TIA) or stroke. Kidney damage or an estimated glomerular filtration rate (eGFR) less than 60 ml/min/1.73 m persisting for three months or more was considered chronic kidney disease (CKD). According to the CDC, patients who were symptom-free for three continuous days and 7 to 10 days had passed since the beginning of their symptoms were considered to have recovered from COVID-19 [20].

### Blood pressure and anthropometric measurements

A doctor or nurse measured blood pressure in the seated position using a validated electronic blood pressure monitor (Omron J30, Omron Healthcare, Kyoto, Japan) in a quiet room after 15 min of rest. Patients were not allowed to drink alcohol, smoke, eat heavy meals, or take antihypertensive drugs before the measurements. To address masked hypertension and white coat hypertension, BP measurements were compared with Home Blood Pressure Monitoring (HBPM) and Ambulatory Blood Pressure Monitoring (ABPM) data in some patients, when available, to reevaluate inconsistencies. An appropriate cuff size was chosen regarding arm circumference. Two measurements were done in each session around the right upper arm within 10 min. The average of the two measurements was used in the analysis. Body height and body weight were self-reported. Body mass index (BMI) was calculated as the body weight in kilograms divided by the body height in meters squared.

### Statistical analysis

Statistical Package for the Social Sciences (SPSS) v. 20 (IBM corporation, USA) was utilized for data analysis. Categorical variables are shown as frequency (percentage), and continuous variables are depicted as mean ± standard deviation (SD). The paired t-test was used to compare before and after COVID-19 systolic and diastolic blood pressures. Linear regression analysis was used to assess the predictors of systolic and diastolic blood pressure changes. P-values < 0.05 were considered statistically significant.

## Results

### Demographics and clinical characteristics

A total of 5,355 patients with a confirmed COVID-19 diagnosis participated in the study, with an average age of 55.51 ± 15.38 years. Of these, 2,510 (46.9%) were male. Table 1 provides a detailed overview of patient demographics, comorbidities, and symptoms during their SARS-CoV-2 infection.

**Table 1:** Demographic and clinical characteristics, n (%) or mean ± SD

	*N* = 5355
Age [mean (SD), years]	55.51 (15.38)
Sex [male (%)]	2510 (46.9%)
BMI [mean (SD), kg/m2]	28.86 (6.28)
Hypertension [*n* (%)]	3045 (56.9%)
Diabetes mellitus [*n* (%)]	1820 (34%)
Cardiovascular event [*n* (%)]	2374 (44.3%)
HLP	472 (8.8%)
CVA [*n* (%)]	160 (3%)
COPD [*n* (%)]	156 (2.9%)
Smoke [*n* (%)]	994 (18.6%)
Alcohol [*n* (%)]	71 (1.3%)
CKD	41 (0.7%)

BMI: body mass index, CVA: cerebrovascular accident, COPD: chronic obstructive pulmonary disease, HLP: hyperlipidemia, CKD: chronic kidney disease

### Comorbidities, and symptoms

The average BMI was 28.86 ± 6.28 kg/m^2. The most prevalent comorbidities included hypertension (56.9%), cardiac events (44.3%), diabetes mellitus (34%), and hyperlipidemia (8.8%). COPD and CKD were less common, observed in 156 (2.9%) and 41 (0.7%) patients, respectively. Additionally, 3% (160) of participants had a history of previous cerebrovascular accidents (CVA). A smaller proportion reported alcohol consumption (1.3%) or smoking (18.6%). During the active phase of COVID-19, fatigue, cough, body pain, and fever were the predominant symptoms. The most commonly prescribed antihypertensive medications were ACE inhibitors, angiotensin receptor blockers (ARBs), and calcium channel blockers.

### Incidence of new or exacerbated hypertension

**Fig. 2:**
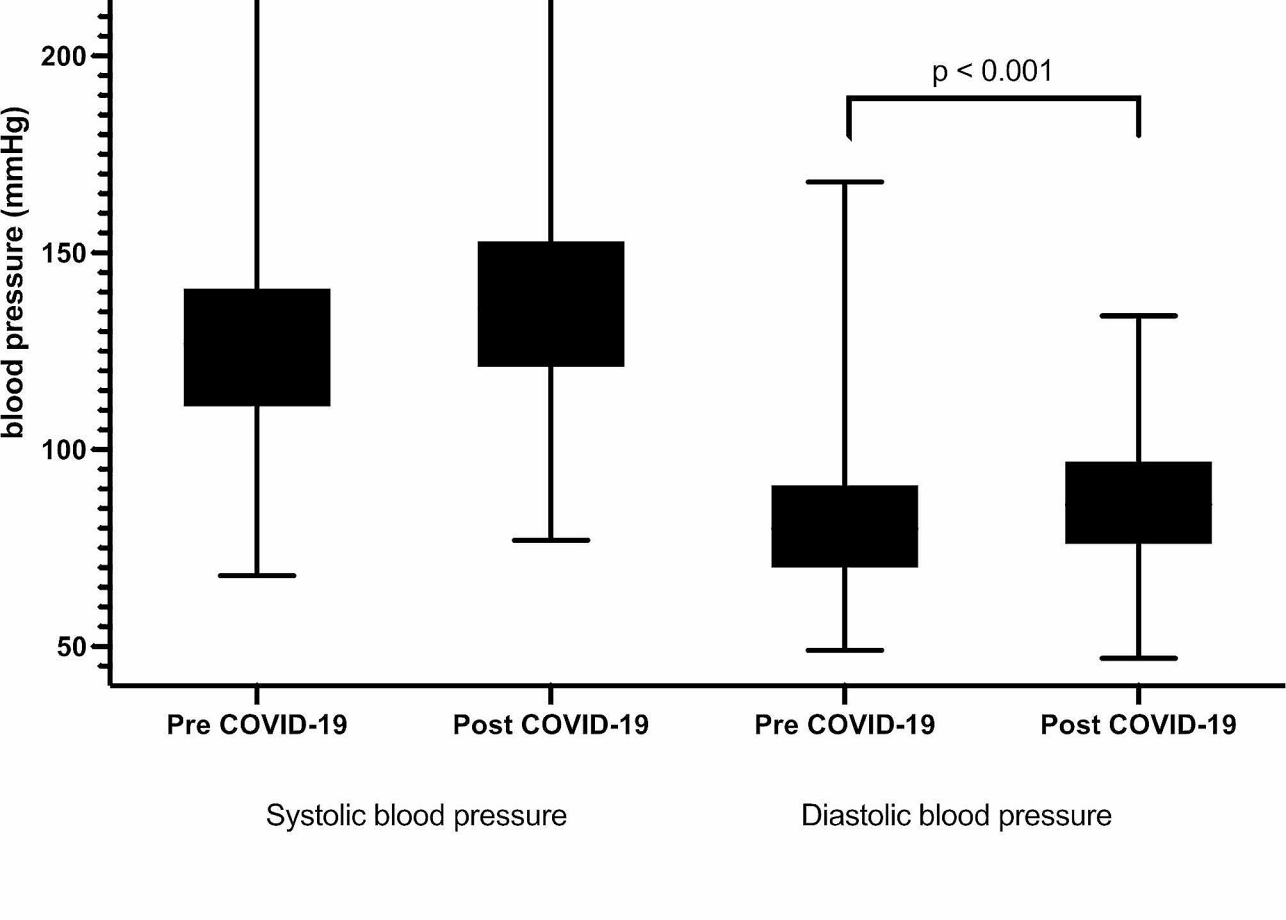
Systolic and diastolic blood pressures before COVID-19 and after recovery from COVID-19

Patients were followed up at the cardiology clinic approximately 12.5 ± 0.4 months after recovering from COVID-19. Approximately 25% of patients had their office blood pressure readings validated using HBPM or ABPM. We observed a significant increase in both systolic and diastolic blood pressure (BP) post-recovery compared to pre-COVID-19 levels (Fig. 2). Specifically, systolic BP increased from 126.90 ± 20.91 mmHg to 139.99 ± 23.94 mmHg (*P* < 0.001), and diastolic BP increased from 80.54 ± 13.94 mmHg to 86.49 ± 14.40 mmHg (*P* < 0.001). A total of 864 patients (16%) developed new or exacerbated hypertension post-recovery. These patients required adjustments to their antihypertensive medications. The mean increase in systolic BP was 10.09 mmHg, which was significantly higher than the mean increase in diastolic BP of 5.94 mmHg (*P* < 0.001) (Table 2).

**Table 2:** Blood pressure before and after COVID-19, mean ± SD

	*N* = 5355	Paired t-test *p*-value
Pre-COVID-19 systolic pressure [mean (SD), mmHg]	126.90 (20.91)	< 0.001
Post-COVID-19 systolic pressure [mean (SD), mmHg]	136.99 (23.94)	
Pre-COVID-19 diastolic pressure [mean (SD), mmHg]	80.54 (13.94)	< 0.001
Post-COVID-19 diastolic pressure [mean (SD), mmHg]	86.49 (14.40)	
Change in systolic pressure [mean (SD), mmHg]	10.09 (29.72)	< 0.001
Change in diastolic pressure [mean (SD), mmHg]	5.94 (19.40)	

### Predictors of blood pressure changes

Age, previous history of hypertension, diabetes mellitus, previous cardiac events, and smoking were identified as significant predictors of BP variations following SARS-CoV-2 infection (*P* < 0.001). Conversely, factors such as gender, BMI, COPD, CVA, CKD, hyperlipidemia, and alcohol consumption did not predict changes in BP post-infection (*P* > 0.05). Table 3 presents the predictors of BP change.

**Table 3:** Predictors of change in systolic and diastolic pressure after COVID-19.

Variable	Systolic BP			Diastolic BP		
Variables	Beta coefficient	Standard error of beta coefficient	*p*-value	Beta coefficient	Standard error of beta coefficient	*p*-value
Age	0.21	0.02	**< 0.001**	0.09	0.01	**< 0.001**
Sex (female vs. male)	0.1	0.81	0.895	-0.13	0.53	0.801
BMI	0.03	0.07	0.614	0.0001	0.04	0.995
HTN	9	0.81	**< 0.001**	3.69	0.53	**< 0.001**
Diabetes	12.52	0.84	**< 0.001**	5.18	0.55	**< 0.001**
Cardiovascular event	12.45	0.8	**< 0.001**	5.45	0.52	**< 0.001**
CVA	-4.05	2.38	0.089	1.16	1.55	0.454
COPD	2.22	2.41	0.357	-2.27	1.57	0.15
HLP	-1.45	1.43	0.312	0.788	0.935	0.400
CKD	2.19	4.66	0.638	2.90	3.04	0.340
Smoking	20.27	1	**< 0.001**	8.33	0.67	**< 0.001**
Alcohol consumption	5.78	3.55	0.103	2.05	2.31	0.377

BMI: body mass index, CVA: cerebrovascular accident, COPD: chronic obstructive pulmonary disease, HLP: hyperlipidemia, CKD: chronic kidney disease

## Discussion

The acute phase of COVID-19 has been explored extensively, including factors affecting its incidence, severity, mortality, and patient survival; however, data about the disease’s sequelae are limited. With the prolongation of the pandemic, the short-term and long-term effects of COVID-19 on different body systems began to move into the research spotlight. In an unprecedented undertaking, we examined the long-term effects of COVID-19 on BP among 5,355 non-hospitalized patients who were followed up 12.5 ± 0.4 months after recovering from COVID-19. Both systolic and diastolic BPs were significantly higher post-infection. There were 456 (17%) patients with new-onset hypertension and 456 (14%) with exacerbated hypertension requiring an intensified antihypertensive regimen. Age, previous history of HTN, DM, or cardiac events, and smoking were the predictors of BP increase.

### Previous studies on COVID-19 and blood pressure

Hypertension, alongside diabetes mellitus and coronary artery disease, has been identified as a prevalent comorbidity in COVID-19 patients, affecting approximately 27–30%, 19%, and 6–8% of cases, respectively [21, 22]. Studies investigating blood pressure variations among COVID-19 patients, primarily focusing on hospitalized individuals over short follow-up periods, have yielded insightful findings. In accordance with the results of the present study, a recent observational cohort study revealed that the incidence of new-onset hypertension significantly rose during the pandemic to 5.20 per 100 person-years, compared to the pre-pandemic rate of 2.11. Remarkably, the trend remains positive even in 2023, post-pandemic termination, suggesting ongoing high levels of virus circulation [23]. Akpek et al. demonstrated increased systolic (120.9 ± 7.2 vs. 126.5 ± 15.0 mmHg) and diastolic (78.5 ± 4.4 vs. 81.8 ± 7.4 mmHg) blood pressure shortly after COVID-19 infection, potentially leading to new-onset hypertension among hospitalized patients [24]. Gameil et al. observed a rise in systolic blood pressure among COVID-19 survivors at three months post-infection compared to gender-matched controls [25]. De Lorenzo et al. reported that a significant portion (58.9%) of discharged COVID-19 patients required ongoing medical care, with uncontrolled hypertension accounting for 21.6% of cases [26]. Nandadeva et al. found no disparity in ambulatory and laboratory-measured blood pressure between COVID-19 patients and controls; however, there was a notable inverse relationship between blood pressure and time since infection, suggesting higher blood pressure levels with more recent disease onset [27]. Chen et al. noted elevated Ang II levels in COVID-19 patients without prior hypertension compared to healthy controls, along with significantly higher systolic blood pressure in the patient group. Moreover, a subgroup of patients developed hypertension post-COVID-19, characterized by heightened Ang II levels compared to non-hypertensive COVID-19 patients [28]. These observations imply that blood pressure fluctuations may stem from hormonal changes following SARS-CoV-2 infections.

### Possible reasons for BP fluctuation following COVID-19

**Fig. 3:**
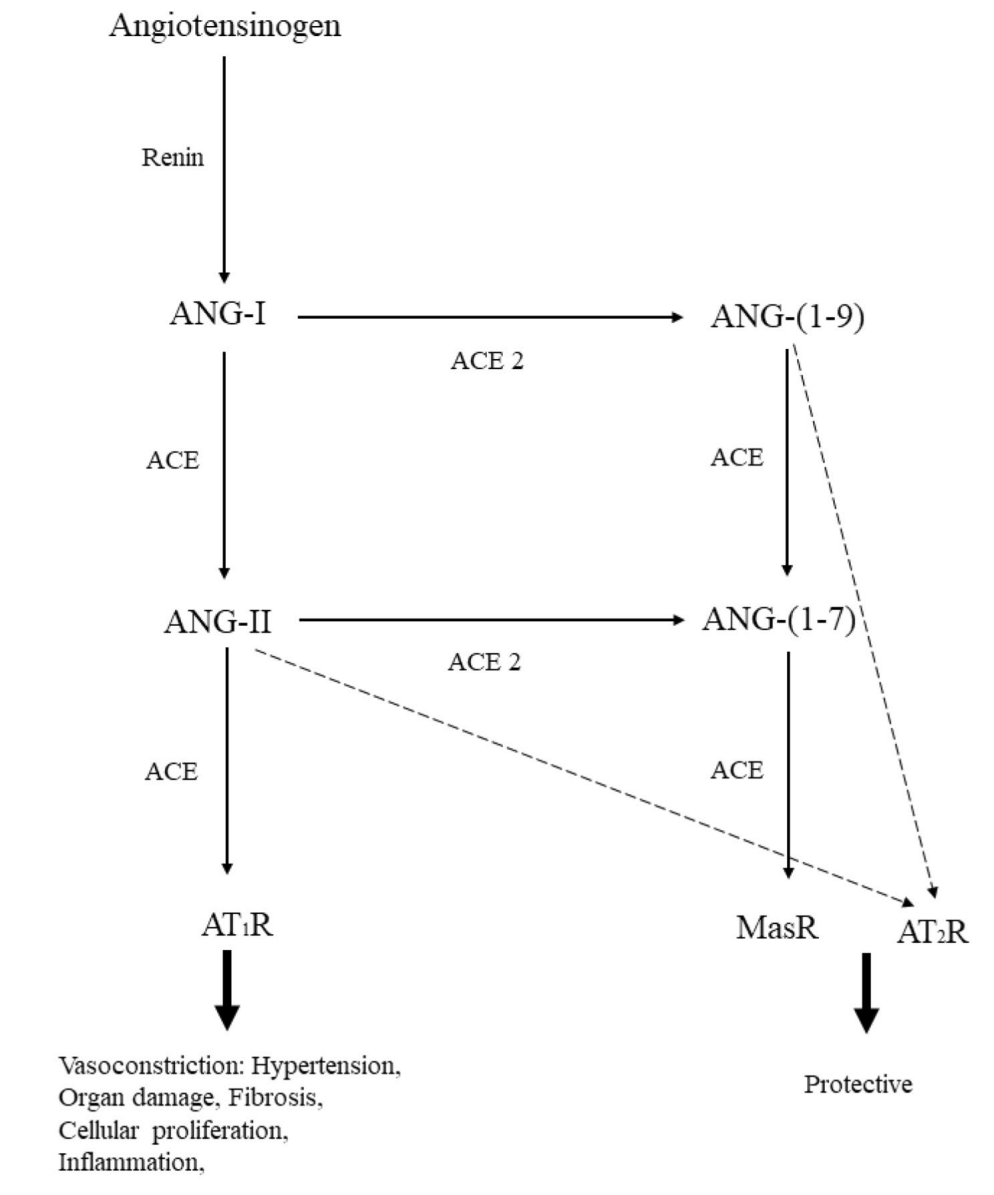
Overview of the RAAS. ANG-I, angiotensin I; ANG-II, angiotensin II; ANG-(1–7), angiotensin-(1–7); ANG-(1–9), Angiotensin-(1–9); ACE, angiotensin converting enzyme; ACE2, angiotensin converting enzyme 2; AT1R, angiotensin type-I receptor; AT2R, angiotensin type-II receptor; MasR, MAS receptor. Adapted from Ravichandran et al. [30]

The renin-angiotensin-aldosterone system (RAAS) plays a pivotal role in regulating blood volume, vascular resistance, and blood pressure [29]. It operates through two primary axes: the angiotensin-converting enzyme/angiotensin-II/angiotensin type-I receptor (ACE/ANG-II/AT1R) and the angiotensin-converting enzyme 2/angiotensin- [1–7]/MAS-receptor (ACE2/ANG- [1–7]/MAS) axes [30]. ACE2, expressed in various tissues such as the heart, intestines, kidneys, testis, and gallbladder, acts not only as a modulator of RAAS but also as the cell entry receptor for both SARS-CoV-1 and SARS-CoV-2 [31]. During the initial stages of the COVID-19 pandemic, there were concerns regarding the use of ACE inhibitors and ARBs due to their potential upregulation of ACE2. This led to speculations that these antihypertensive drugs might increase the susceptibility and severity of COVID-19 [32]. However, subsequent reviews and cohort studies dismissed this hypothesis, affirming the safety of these medications and even suggesting potential protective effects against COVID-19-related complications [33, 34]. 2 Additionally, ADAM17 can cleave the extracellular juxta-membrane region of ACE2, leading to an increase in the soluble form of ACE2. This soluble isoform may compete with membrane-bound ACE2, potentially preventing viral entry into cells [35, 36]. SARS-CoV-2 enters host cells by binding to ACE2 and TMPRSS2, leading to a transient downregulation of ACE2 and disrupting the balance between ACE2 and angiotensin II [37–39]. This disruption can increase ANG II, resulting in increased vasoconstriction, aldosterone release, and fluid retention, contributing to elevated blood pressure [40–42]. Moreover, this peptide hormone causes an increase in endothelial and organ damage by producing reactive oxygen species (ROS) [43]. Furthermore, Human recombinant soluble ACE2 (hrsACE2) has emerged as a potential therapeutic candidate for COVID-19 due to its ability to bind SARS-CoV-2 and potentially reduce viral entry into host cells [44]. Reduced ACE2 levels may also downregulate the protective ACE2/ANG- [1–7]/MAS axis. This axis has been shown to protect against conditions such as lung fibrosis and inflammation [45–47] In summary, the dysregulation of RAAS, characterized by increased angiotensin II levels, sodium and water retention, vasoconstriction, and downregulation of protective pathways, may contribute to elevated blood pressure in COVID-19 patients (Fig. 3) [48].

In addition to ARDS and AKI, SARS-CoV-2 can also result in complications affecting other systems, including cardiovascular issues [30]. Increasing evidence suggests that SARS-CoV-2 has a systemic impact, causing vascular damage and dysfunction, particularly affecting the endothelium and potentially linking various organs and systems [49, 50]. The virus can indirectly affect microvascular endothelial cells, leading to changes in endothelial functions. Oxidative stress initiates endothelial damage [51, 52], exacerbating alterations in cell permeability. Additionally, modifications in the expression of adhesion molecules and receptors related to angiogenesis can lead to an increased neutrophil count, the formation of neutrophil extracellular traps, inflammation, blood clotting, and reduced oxygen levels [53]. The virus has been detected in the myocardium during autopsy analysis [54, 55], and it has been reported to induce endotheliitis, myocardial ischemia, and arrhythmia by penetrating the small blood vessel walls in the heart [56, 57]. Furthermore, the disruption of arteriolar endothelial walls, crucial for regulating blood flow and vascular resistance, contributes to blood pressure variations following infection [58]. However, the duration and long-term impact of these disturbances on the cardiovascular system in patients remains uncertain.

Concerns regarding the safety of SARS-CoV-2 vaccines have arisen due to reported incidents of thromboembolic events, hypersensitivity reactions, and tachycardia post-vaccination [43, 59, 60]. Additionally, reports have highlighted BP elevation following COVID-19 vaccination. In a systematic review and meta-analysis by Angeli et al., encompassing six studies and 357,387 subjects, incidence rates of abnormal/increased BP and stage III hypertension/hypertensive urgencies and emergencies were found to be 3.20% and 0.6%, respectively [61]. Meylan et al. documented instances of stage III hypertension in nine patients shortly after mRNA-based SARS-CoV-2 vaccination, with eight having a history of hypertension; all recovered following monitoring and antihypertensive treatment [43]. These findings are supported by Zappa et al., who observed an average increase in systolic or diastolic BP by ≥ 10 mmHg at home during the first five days after the initial vaccine dose, particularly among individuals with a prior COVID-19 history [62]. Moreover, the vaccine recipients with pre-existing immunity exhibited a higher frequency and severity of systemic reactions compared to those without immunity [63]. This observed increase in BP post-vaccination suggests a potential association with elevated BP post-COVID-19 infection, possibly linked to RAAS overactivation.

Immune system activation and subsequent inflammation are determinants of systolic, diastolic, and pulse pressure in COVID-19 patients [64, 65]. Inflammatory cytokines released by viral and bacterial infections cause microvascular dysfunction, disrupting kidney microcirculation and potentially leading to BP variation [66]. The role of inflammation in aortic stiffness and BP has been confirmed in previous studies; it induces endothelial injuries and dysregulation, reducing nitric oxide production [67]. This vasodilator is a promising remedy in the treatment of HTN, but its bioavailability decreases in COVID-19 patients [49]. A randomized clinical trial indicated that the immune response against pathogens increases aortic stiffness—a pivotal basis of systolic BP [68]. Incubation of COVID-19 serum has been shown to induce endothelial damage, including apoptosis and impaired angiogenic capacity as well as triggering thrombotic events [13, 69].

Despite the present study evaluating non-hospitalized patients, some hospitalized COVID-19 may experience prolonged periods of mechanical ventilation, associated sedation, volume overload, inotropic use, increased adrenergic tone, fever, hypoxia, inflammation, cytokine storm, ischemia, and vasculitis. These factors may contribute to an excessive cardiovascular response and worsening of hypertension [7]. ICU-admitted COVID-19 patients tend to develop hyperreninemia combined with hypernatremia and hyperchloremia [70]. It has been also indicated that three months after hospitalization due to COVID-19, patients experience sympathetic neural overdrive, vascular dysfunction, increased arterial stiffness, and reduced exercise capacity. These factors may contribute to clinical manifestations such as elevated blood pressure [15, 71].

Blood pressure changes over a long period are influenced by quite diverse factors; besides the effect of COVID-19, other factors may change the BP during the pandemic indirectly. According to studies worldwide, lockdowns have been linked with several adverse long-term effects on cardiovascular diseases due to increased stress, anxiety, isolation, obesity, and decreased physical activity [72, 73]. Evaluating these factors was not the purpose of the present study, but their effects cannot be ignored. According to Di Renzo et al., outdoor physical activities such as walking, jogging, and swimming decreased significantly during the pandemic [74]. In a survey in Vietnam, 3.3% and 2.6% of participants reported a reduction and cessation of exercise during the pandemic lockdown, respectively. Moreover, 12.4% of patients had difficulty controlling their BP; a sedentary lifestyle, decreased medication adherence, and inaccessibility of medical facilities could be possible etiologies [75]. The pandemic has had a significant impact on the continuity of care, medication adherence, and blood pressure management among individuals with hypertension, exacerbating challenges in achieving optimal blood pressure control. Pandemic-related disruptions, such as lockdowns and healthcare system strain, have led to interruptions in medication adherence, as evidenced by a decline from 86.0 to 80.8% according to a retrospective cohort study involving 64,766 individuals. Concurrently, in-person primary care visits decreased from 2.7 to 1.4 per year during the pandemic [76]. In the present study, no patients experienced remote BP management. Despite this, as a result of the pandemic’s disruption to traditional healthcare services, there has been a notable increase in the adoption of remote management programs aimed at addressing the widespread issue of poorly controlled hypertension [77]. This shift towards remote management has shown promising results; notably, the proportion of patients with sustained hypertension reaching their blood pressure goals increased from 75.8 to 94.6% during the pandemic period, facilitated by remote monitoring and frequent patient-provider communication [78]. Moreover, maintenance of blood pressure was achieved earlier during the pandemic, indicating the effectiveness of remote management strategies coupled with enhanced patient support and engagement.

### Possible risk factors for blood pressure changes following COVID-19

**Fig. 4:**
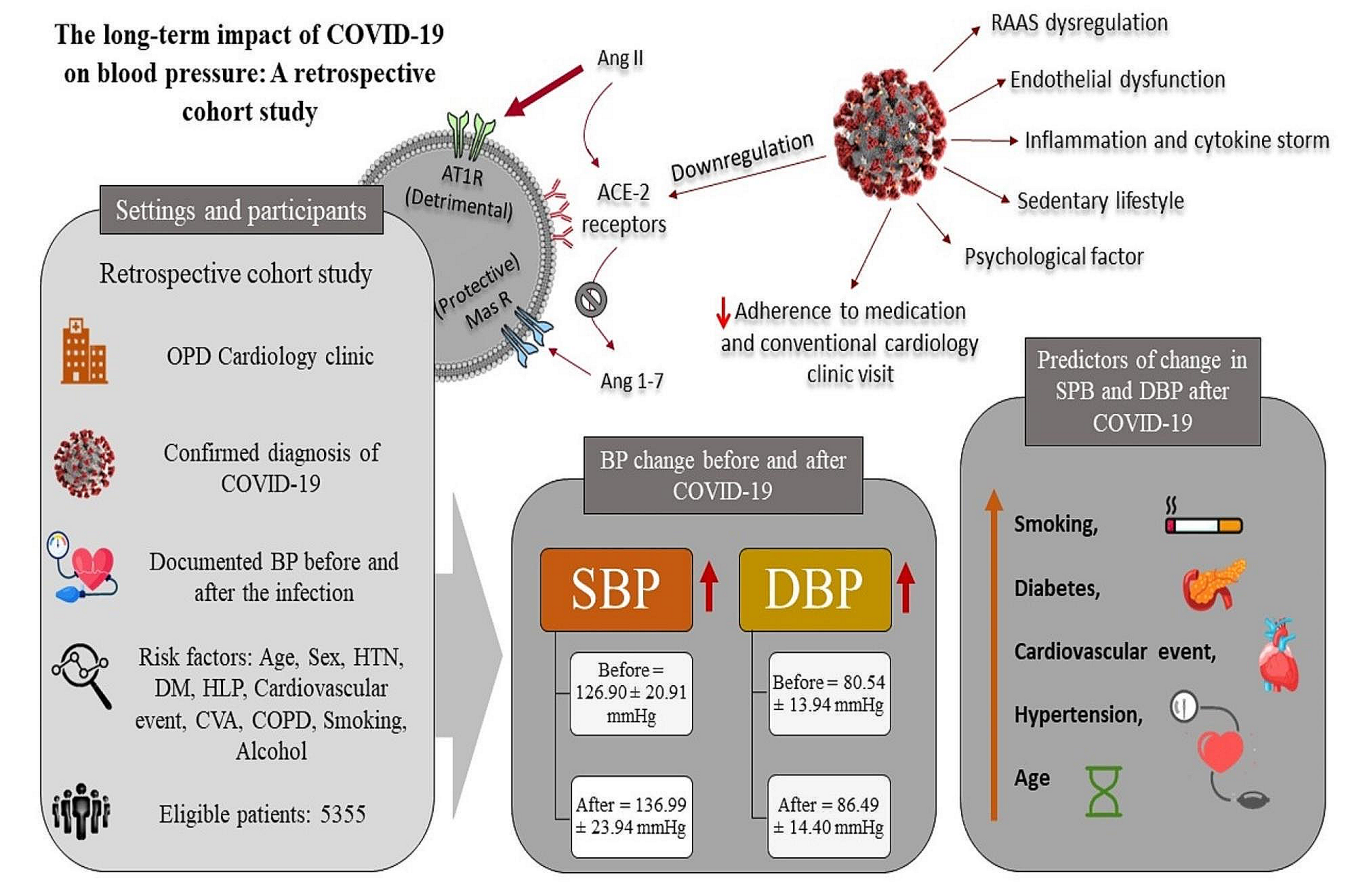
Main graphical abstract, showing the possible relation between Covid 19 infection and hypertension

Our investigation suggested that patients with older age and comorbidities (including HTN, DM, smoking, and a history of cardiovascular events) are at higher risk of BP increase after COVID-19 recovery. Previous meta-analyses and observational studies cite these characteristics as predictors of COVID-19 severity and mortality [79, 80]. It was suggested that the upregulation of fibroblasts in COVID-19 patients with HTN and DM may contribute to lung fibrosis and a decline in lung function [81]. RAAS dysregulation (decreased ACE 2 and Ang 1–7; increased Ang II) can potentially explain the abovementioned associations. Endothelial dysfunction in patients with hypertension, diabetes, obesity, or aging, combined with vascular damage induced by COVID-19, has been linked to severe morbidity and mortality. This suggests that the direct effects of SARS-CoV-2 on the vascular system may amplify the mortality risk in COVID-19 patients [82]. Age, history of hypertension, diabetes, and previous cardiac events were predictors of uncontrolled hypertension in the COVID-19 group in the Angeli study [19]. Similarly, Nam et al. associated advanced age and a history of hypertension with increased BP variability [83]. Thus, comorbidities such as DM and HTN must be controlled to prevent a severe COVID-19 course and subsequent sequelae. (Fig. 4, graphical abstract)

### Limitations and strengths

The current study has some limitations. First, due to the retrospective nature of the study, we could not establish a control group to compare BP levels between cases and controls, which limits our ability to draw causal conclusions about the observed variations in BP. Additionally, the retrospective design means controlling for confounding factors is challenging, making it difficult to distinguish whether the fluctuations in blood pressure are directly related to the pathophysiological effects of the virus or indirectly influenced by factors prevalent during the COVID-19 era, such as a sedentary lifestyle, psychological factors, or decreased adherence to treatment. Information bias may be present due to reliance on medical records, potentially introducing inaccuracies or omissions in recorded data. Moreover, recall bias might affect the accuracy of reported information about medication and past medical history, as it relies on patients’ recall of past events and medication details. Second, the BP levels were measured at the clinic, and ambulatory daytime, nighttime, and 24-hour BP measurements were unavailable for the majority of the patients. While such measurements would provide a more comprehensive assessment, the likelihood of white-coat hypertension influencing our results seems minimal. BP was measured in a quiet room under comfortable conditions, and initial and final measurements from the same individual were compared using paired statistical analysis, enhancing the reliability of our findings. Third, serial BP measurements across multiple follow-up sessions would have enabled us to determine the persistence of any observed changes over time. Fourth, this study was conducted at a single center, representing a limited racial, ethnic, and geographical area, which may limit the generalizability of the findings. On the other hand, the large sample size of non-hospitalized patients is a significant strength of this study, potentially offering insights that are more representative of the broader population affected by COVID-19 compared to studies focusing solely on hospitalized patients. Another strength is that BP measurements were not taken during the acute phase of COVID-19, eliminating the potential confounding effects of acute disease processes and related psychological factors on BP readings. Nonetheless, multicenter prospective studies with longer follow-up periods are warranted to overcome the limitations of the present work and provide more robust evidence.

## Conclusion

Our findings suggest a potential association between COVID-19 and increased systolic and diastolic BP in non-hospitalized patients, with a notable proportion of participants experiencing new-onset or exacerbated hypertension. While the precise mechanisms remain to be fully understood, possible contributors may include RAAS dysregulation, inflammatory processes, systemic cytokine responses, as well as lifestyle and psychological factors such as sedentary behavior, stress, and anxiety post-COVID-19. Additionally, older patients, smokers, individuals with comorbidities like HTN and DM, and those with a history of MACE may be more vulnerable to BP changes following COVID-19. Given these findings, regular BP monitoring is advised for patients recovering from COVID-19, especially for those with the identified risk factors.

## Data Availability

All data are available in professor Kojuri cardiology clinic, registry and available. with reasonable request.

## Abbreviations

ACE2: Angiotensin converting enzyme 2
ACS: Acute coronary syndrome
ABPM: Ambulatory Blood Pressure Monitoring
SARS-CoV-2: Severe Acute Respiratory Syndrome Corona Virus 2
CABG: Coronary artery bypass graft
CAD: Coronary artery disease
CP: Chest pain
CVD: Cardiovascular diseases
DM: Diabetes mellitus
DOE: Dyspnea on exertion
HF: Heart failure
HLP: Hyperlipidemia
HBPM: Home Blood Pressure Monitoring
HRCT: High-Resolution Computed Tomography
HTN: Hypertension
ICU: Intensive care unit
MACE: Major adverse cardiovascular events
MI: Myocardial infarction
PCR: Polymerase chain reaction
PCI: Percutaneous coronary intervention
PND: Paroxysmal nocturnal dyspnea
WHO: World Health Organization
